# Embryonic Exposure to Valproic Acid Affects Social Predispositions for Dynamic Cues of Animate Motion in Newly-Hatched Chicks

**DOI:** 10.3389/fphys.2019.00501

**Published:** 2019-04-30

**Authors:** Elena Lorenzi, Alessandra Pross, Orsola Rosa-Salva, Elisabetta Versace, Paola Sgadò, Giorgio Vallortigara

**Affiliations:** ^1^Center for Mind/Brain Sciences, University of Trento, Rovereto, Italy; ^2^Department of Biological and Experimental Psychology, School of Biological and Chemical Sciences, Queen Mary University of London, London, United Kingdom

**Keywords:** valproic acid, social predispositions, newly-hatched chick, autism spectrum disorder, animacy, *Gallus gallus*

## Abstract

Early predispositions to preferentially orient toward cues associated with social partners have been documented in several vertebrate species including human neonates and domestic chicks. Human newborns at high familiar risk of Autism Spectrum Disorder (ASD) show differences in their attention toward these predisposed stimuli, suggesting potential impairments in the social-orienting mechanisms in ASD. Using embryonic exposure to valproic acid (VPA) we modeled ASD behavioral deficits in domestic chicks. To investigate social predispositions toward animate motion in domestic chicks, we focused on self-propulsion, using two video-animations representing a simple red circle moving at constant speed (speed-constant) or one that was changing its speed (accelerating and decelerating; speed-change). Using a spontaneous choice test for the two stimuli, we compared spontaneous preferences for stimuli that autonomously change speed between VPA- and vehicle-injected chicks. We found that the preference for speed changes was abolished in VPA-injected chicks compared to vehicle-injected controls. These results add to previous findings indicating similar impairments for static social stimuli and suggest a specific effect of VPA on the development of mechanisms that enhance orienting toward animate stimuli. These findings strengthen the hypothesis of an early impairment of predispositions in the early development of ASD. Hence, early predispositions are a potentially useful tool to detect early ASD symptoms in human neonates and to investigate the molecular and neurobiological mechanisms underlying the onset of this neurodevelopmental disorder.

## Introduction

Neonates of some vertebrate species orient their first approach responses toward objects that exhibit features present in social partners and caregivers: face-like configuration, biological motion and self-propulsion. Comparative research on human infants and newly hatched domestic chicks (*Gallus gallus*) found striking similarities in the static and dynamic visual cues that attract attention of these different species soon after birth (Di Giorgio et al., 2017a). Among dynamic cues, point-light displays depicting biological motion are preferred by neonates of both species to the same configuration of dots rigidly rotating or moving randomly (Vallortigara and Regolin, 2006; Simion et al., 2008). Chicks also seem to have a spontaneous preference for objects autonomously starting to move over objects set in motion after a collision (Mascalzoni et al., 2010) and for objects autonomously changing their speed over constant moving ones (Rosa-Salva et al., 2016). Similarly, human neonates exhibit a looking preference for self-propelled objects autonomously starting from rest (Di Giorgio et al., 2017b).

Alterations in social predispositions appear to be linked to Autistic Spectrum Disorders (ASD) a complex group of neurodevelopmental disabilities characterized by important deficits in the domain of social cognition (Sacrey et al., 2015). Impairments in face discrimination and recognition have been widely observed in ASD individuals (Dawson et al., 2005). Young children with ASD show altered processing of stimuli depicting biological motion (Freitag et al., 2008; Klin et al., 2009) and difficulties in spontaneous categorization of self-propelled motion as animate (Rutherford et al., 2006). Neonates at high familiar risk of ASD show significant differences compared to low-risk neonates in the preference for a face-like stimulus and for biological motion, suggesting an impairment in the development of the predisposed mechanisms for detecting animate beings (Di Giorgio et al., 2016). Observing the same impairment for both static and dynamic stimuli in a different species would argue in favor of a common developmental origin of these predispositions.

Valproic acid is an anticonvulsant and a mood stabilizer widely used to treat epilepsy, migraine and bipolar disorder (Johannessen and Johannessen, 2003). In humans, prenatal exposure to VPA has been shown to increase the risk of developing ASD (Christensen et al., 2013). Embryonic exposure to VPA has been widely used to model the ASD syndrome in rodents (see for a review Nicolini and Fahnestock, 2018). Embryonic exposure to VPA has been shown to induce impairments in chicks’ aggregative behavior (Nishigori et al., 2013) and in their early predisposition for static stimuli (Sgadò et al., 2018).

To further study the effect of VPA on early predispositions, and to investigate whether the impairment for static cues is accompanied by impairment in predispositions for dynamic cues, we compared the spontaneous preference for self-propelled stimuli in VPA- and vehicle-injected chicks.

## Materials and Methods

### Embryonic Injections

Fertilized eggs of domestic chicks (*Gallus gallus*) of the Ross 308 (Aviagen) strain were obtained from a local commercial hatchery (Agricola Berica, Montegalda, Italy) and incubated at 37.7°C and 60% of relative humidity in the darkness. The first day of incubation was considered embryonic day 0 (E0). At E14, fertilized eggs were selected by candling before injection. Embryo injection was performed according to previous reports (Nishigori et al., 2013; Sgadò et al., 2018). Briefly, a small hole was made on the eggshell above the air sac, and 35 μmoles of VPA (Sodium Valproate, Sigma-Aldrich) dissolved in double distilled injectable water were administered to each fertilized egg, in a volume of 200 μl. Age-matched control eggs were injected using the same procedure with 200 μl of vehicle (double distilled injectable water). After sealing the hole with paper tape, eggs were placed back in the incubator (FIEM srl, Italy). Previous reports have analyzed the effect of different doses and time of administration of VPA on embryonic development in different vertebrate species (see for a review Roullet et al., 2013; Ranger and Ellenbroek, 2016). The typical dose and time of administration in rodents is 200–500 mg/kg in acute, single dose administration between E12 and E14. In domestic chicks, administration of 35 μmoles/egg (corresponding to approximately 100 mg/kg) has been tested between E10 and E14 with differential effects on hatching rate, showing a dramatic decrease of hatchings at E10 and a significant decrease of hatchings at E12 but no significant effect at E14 (Nishigori et al., 2013). Administration of 35 μmoles/egg at E14 induced social deficits without affecting hatchability, motor behavior and imprinting abilities (Nishigori et al., 2013; Sgadò et al., 2018).

During incubation and hatching, eggs and chicks were maintained in complete darkness, preventing any visual experience prior to the test. Controlling the visual experience during pre- and post-natal development enable to exclude any interference of visual stimuli in the expression of predispositions toward animacy cues, and to demonstrate the innate nature of these mechanisms. Each chick was tested only once.

### Apparatus, Stimuli and Test

We used the same procedure previously described to assess chicks’ predispositions for speed-changes. Briefly, carefully avoiding any other visual experience, the day of hatching chicks were individually placed in the center of the test apparatus, a corridor (85 × 30 × 30 cm), open at the two ends where two video screens were displaying the experimental stimuli. The corridor was divided in three sectors: a central sector (45 cm long) delimited by two steps, that the animals had to climb to enter the two choice sectors (each 20 cm long) immediately adjacent to the two screens. Stimuli were two video-animations representing the movement of a simple red circle. In one video the object was moving at constant speed (speed-constant) and in the other one it was changing its speed (accelerating and decelerating; speed-change). A spontaneous choice test of 6 min was performed for the two stimuli. Chicks’ preference for the speed-change stimulus was measured by the ratio of time (in seconds) spent in the choice sector near the speed-change stimulus divided by the cumulative time spent in either of the choice sectors (preference). Chicks remaining in the central sector were not included in the analyses. Values of this ratio could range from 0 (full preference for the speed-constant), to 1 (full preference for the speed-change), whereas 0.5 represented no preference. For more detailed information on the procedure, see Rosa-Salva et al. (2016). Chicks’ level of motility was measured by evaluating the latency (in seconds) to first approach, irrespective of the stimulus approached. The tests were performed manually and scored online. To evaluate reliability of scoring and potential biases, 10% of all subjects were scored again offline by a second experimenter blind to the treatment group and right/left position of the two stimuli. Overall, we blindly coded videos of 10 animals randomly chosen from both treatment groups. We obtained a Pearson’s correlation of 1.000, *p* < 0.001 between the preference scores calculated using our original data and the blind coding. For the present study 51 VPA-injected (males = 27) and 52 vehicle-injected (males = 26) chicks were tested.

### Data Analysis

Effects of Treatment (VPA and vehicle injection) and Sex (male, female) on the preference for the speed-change stimulus were assessed by a multifactorial analysis of variance (ANOVA) on the dependent variable preference score. One-sample two-tailed *t*-tests were run to test significant departures from chance level (0.5) of the preference score, separately for the two groups. The number of chicks that first approached the speed-change or the speed-constant stimulus in the two treatment groups was compared using the chi-square test of independence. Effects of Treatment and Sex on latency to first approach were assessed by an ANOVA on the latency to first approach one of the stimuli. All statistical analyses were performed with IBM SPSS Statistic for Windows (**RRID:SCR_002865**). Alpha was set to 0.05 for all the tests. The dataset generated for this study is available in Supplementary Table S1 of the Supplementary Material.

## Results

**FIGURE 1:**
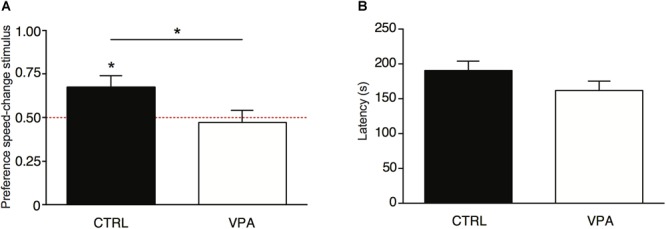
**(A)** Social preference responses for the speed-change stimulus shown as the ratio of time (in seconds) spent in the choice sector near the speed-change stimulus divided by the cumulative time spent in either of the sectors (see section “Materials And Methods” for details). Analysis of variance of social preference scores using Treatment and Sex as between-subjects factors, revealed a significant main effect of Treatment (line with ^∗^), with no other main effects or interactions. Preference scores were significantly different from chance level for vehicle-injected chicks (control group), but not for VPA-treated chicks. Asterisks on top of bars indicate significant departures from chance level, marked by the red line at 0.5. **(B)** Latency to first approach assessed as a measure of motility. Analysis of variance on latency to first approach using Treatment and Sex as between-subject factors showed no significant main effects of Treatment, Sex or interaction Treatment × Sex. Data represent mean ± SEM, ^∗^*p* < 0.05.

The average egg hatchability was 75%. Results of the ANOVA on the preference for the speed-change stimulus showed a significant effect of Treatment [*F*_(1,99)_ = 4.296, *p* = 0.041; Figure 1A], and no significant effect of Sex [*F*_(1,99)_ = 0.0001, *p* = 0.992] nor any significant interaction [Treatment × Sex: *F*_(1,99)_ = 0.151, *p* = 0.698]. In the control group (vehicle-injected), the preference for approaching the speed-change stimulus was similar to what previously observed, and the preference scores were significantly higher than chance level [*t*_(51)_ = 2.365, *p* = 0.011; *M* = 0.673, *SEM* = 0.066, Figure 1A]. On the contrary, VPA exposure significantly reduced the preference for the speed-change stimulus: the preference scores for approaching the speed change stimulus did not differ from chance level [*t*_(50)_ = -0.406, *p* = 0.686; *M* = 0.472, *SEM* = 0.696, Figure 1A]. A significant difference between the two groups was found also in the number of chicks that first approached the speed-change stimulus (χ^2^= 4.314, *p* = 0.047). While in the vehicle-injected group a significantly higher number of chicks first approached the speed-change stimulus (χ^2^= 6.231, *p* = 0.018; speed-change *N* = 35, speed-constant *N* = 17), in the VPA-treated group no significant difference was found in the number of chicks that approached the two stimuli (χ^2^= 0.176, *p* = 0.78; speed-change *N* = 24, speed-constant *N* = 27).

To evaluate motility, we measured the latency to the first approach, independent of the stimulus, and found no significant effects of Treatment [*F*_(1,99)_ = 2.672, *p* = 0.105; Figure 1B], Sex [*F*_(1,99)_ = 1.124, *p* = 0.292], nor any interaction [*F*_(1,99)_ = 0.000, *p* = 0.99].

## Discussion

We investigated unlearned predispositions to orient toward animate motion cues in VPA-injected chicks compared to vehicle-injected controls, using a choice preference test between a speed-change and a constant moving stimulus. We showed a detrimental effect of VPA on the typical spontaneous preference for the speed-change stimuli conveying animacy cues (Rosa-Salva et al., 2016). These results are in line with previous studies investigating static cues to animacy (such as the head and neck region of the mother hen, Sgadò et al., 2018) and our hypothesis of a disruption of unlearned predispositions in animal models of ASD.

In phylogenetically distant species of vertebrates, such as domestic chicks and humans, similar mechanisms have been described to drive early approach responses toward static and dynamic cues typically associated with animate figures. The adaptive function of early predispositions has been hypothesized to be in directing attention toward highly important animate stimuli, enabling future learning through experience and enhancing social interactions (Johnson et al., 2015; Di Giorgio et al., 2017a; Powell et al., 2018). In chicks, predispositions are likely to orient the young animal toward the mother hen (or other brood mates), directing subsequent filial imprinting responses toward animate stimuli (Miura and Matsushima, 2016). In human newborns, subcortical fast and automatic mechanisms have been hypothesized to underlie these social predispositions, directing attention toward animate entities to create an early social bond with the caretakers and social companions (Tomalski et al., 2009; Johnson et al., 2015; Di Giorgio et al., 2017a). Subsequently, experience may modulate and specialize more sophisticated mechanisms devoted to the processing of social stimuli (Johnson et al., 2015; Versace et al., 2016, 2018).

Several accounts suggest that abnormalities in this early social-orienting system may lead to deficits in social stimuli processing, limiting attention to salient social stimuli, decreasing their reward value and resulting in the atypical social behavior associated with ASD.

To investigate the contribution of these social-orienting mechanisms in atypical social behavior related to ASD, we modeled ASD-like social impairments in domestic chicks using embryonic exposure to VPA. We then measured preference responses to different social stimuli, either stationary (the face-like configuration visible in a stuffed hen, Sgadò et al., 2018) or dynamic (speed-changes, this work), in visually-naiïve VPA- injected and vehicle-injected domestic chicks.

In this study, we have investigated social predispositions toward animate motion, focusing on the predisposition to approach objects that appear self-propelled due to an “internal energy source” that produces changes of speed. Using behavioral responses to visual stimuli, we have documented the absence of the typical predisposed preferences for animacy stimuli in domestic chicks, as a consequence of embryonic VPA exposure. This drug has been used to model ASD core deficits in other vertebrate species (Ranger and Ellenbroek, 2016) although chicks are the first precocial species in which its effect on social behavior has been investigated (Nishigori et al., 2013; Sgadò et al., 2018). Precocial species, like domestic chicks, are characterized by the early maturation of the motor and sensory system, that allows to perform behavioral tests soon after birth, before gaining any social experience. Our findings, hence, open new possibilities to tackle the early onset of predispositions relevant for social life, focusing on dynamic cues.

Moreover, these findings extend previous literature reporting impairments in the preference response for static, face-like configurations of the stuffed hen stimulus (Sgadò et al., 2018). The observation of a parallel impairment in social predispositions for both static and dynamic cues in different species suggests a common developmental origin of this social-orienting system. Since the neuroanatomical substrates of predispositions for approaching static and dynamic stimuli are at least partially different (Mayer et al., 2016a,b, 2017; Lorenzi et al., 2017), observing here the impairment of both classes of predispositions suggests the existence of a common mechanism.

Our work on VPA-mediated impairment of early predispositions, together with the deficits documented in human neonates at high risk of ASD (Di Giorgio et al., 2016), supports the hypothesis of early social orienting mechanisms shared across species whose impairment or delay might have a pivotal role in the pathogenesis of autism.

Future studies should capitalize on these findings to investigate the molecular and neurobiological mechanisms underlying those ASD early symptoms that are associated with predisposed orienting mechanisms toward social stimuli.

## Ethics Statement

This study was carried out in accordance with the recommendations of the Italian and European Union laws for the ethical treatment of animals. The protocol was approved by the Ethical Committee of the University of Trento and licensed by the Italian Health Ministry (permit number 986/2016-PR).

## Author Contributions

PS, EV, OR-S, and GV conceived and designed the experiments. EL and AP conducted the experiments. PS, EV, and OR-S developed the behavioral paradigms. EL, AP, PS, OR-S, and EV analyzed the data. EL and PS drafted the manuscript. All the authors wrote the manuscript and approved the final version for publication.

## Conflict of Interest Statement

The authors declare that the research was conducted in the absence of any commercial or financial relationships that could be construed as a potential conflict of interest.

## Supplementary Material

TABLE S1 Dataset table. List of all data generated and analyzed in this study.
